# Serum Asprosin and Peptide Tyrosine Tyrosine (PYY) Levels in Bipolar Disorder

**DOI:** 10.3390/jcm14031012

**Published:** 2025-02-05

**Authors:** Nilifer Gürbüzer, Elif Özcan Tozoğlu, Alev Lazoglu Ozkaya, Filiz Mercantepe

**Affiliations:** 1 Department of Psychiatry, Erzurum Faculty of Medicine, University of Health Sciences, Erzurum 25240, Türkiye; nilifer.gurbuzer@sbu.edu.tr (N.G.); elif.ozcantozoglu@sbu.edu.tr (E.Ö.T.); 2 Department of Biochemistry, Erzurum City Hospital, Erzurum 25240, Türkiye; alev.lazogluozkaya@saglik.gov.tr; 3 Department of Endocrinology and Metabolism, Faculty of Medicine, Recep Tayyip Erdogan University, Rize 53100, Türkiye

**Keywords:** bipolar disorder, asprosin, peptide tyrosine tyrosine, energy homeostasis

## Abstract

**Objective:** In our study, we aimed to investigate the differences in metabolic parameters, serum asprosin and peptide tyrosine tyrosine (PYY) levels in a bipolar disorder manic (BD-M) group, a euthymic group and in healthy controls; we also aimed to evaluate the relationship of asprosin and PYY levels with metabolic parameters and psychopathology in patients. **Methods:** The study included 54 manic patients, 40 euthymic patients and 39 healthy controls. The sociodemographic characteristics of the participants were recorded, and biochemical parameters and asprosin and PYY levels were measured. The Young Mania Rating Scale (YMRS) and the Hamilton Depression Rating Scale (HAM-D) were completed. **Results**: Body mass index (BMI) showed significant differences between the three groups (*p* < 0.001); the lowest was found in the control group and the highest in the euthymic group. Triglyceride levels were significantly higher in the euthymic group compared with the BD-M group and controls (*p* = 0.003). Glucose levels were significantly higher in the BD-M group compared with euthymic (*p*_manic-euthymic_ = 0.008) and controls (*p*_manic-control_ < 0.001). Asprosin (*p*_manic-control_ < 0.001, *p*_euthymic-control_ = 0.046, *p*_manic-euthymic_ = 0.015) and PYY (*p*_manic-control_ < 0.001, *p*_euthymic-control_ = 0.037, *p*_manic-euthymic_ = 0.002) levels were significantly different between the three groups, with the lowest levels in the BD-M group and the highest levels in the control group. The eta squared = 0.18 for asprosin and 0.21 for PYY. In the BD-M group, a moderate negative correlation was found between YMRS and asprosin (*r* = −0.345; *p* = 0.011) and PYY (*r* = −0.376; *p* = 0.005) levels. ROC analysis results showed that asprosin and PYY could be used to predict the manic period in BD-I (AUC_asprosin_:0.775; AUC_PYY_:0.760). After adjusting for asprosin as a covariate using ANCOVA, the difference in PYY between groups remained significant (manic–euthymic groups, *p* = 0.040; manic–control groups, *p* = 0.013). **Conclusions:** The study results revealed that asprosin and PYY levels were low, and metabolic parameters were impaired in the patients. Low asprosin and PYY levels may be indicators of impaired energy homeostasis in BD-I. PYY may be a state marker for manic episodes.

## 1. Introduction

Bipolar disorder (BD) is a chronic, recurrent mental disorder with fluctuations in mood such as mania, hypomania and depression, which usually leads to functional and cognitive impairment and decreased quality of life [1,2]. Metabolic imbalances, including overweight, impaired lipid profile, diabetes, metabolic syndrome and obesity are commonly observed in BD [3,4]. Epidemiological and clinical studies have shown that more than half of BD patients are overweight or obese [3]. It has been reported that increased weight may be associated with BD independent of treatment with antipsychotics and mood stabilisers [5]. There is evidence that obesity and metabolic imbalances are associated with adverse outcomes such as resistance to treatment, increased number of mood episodes and increased number of hospitalisations as well as chronicisation of the disease in patients with BD [6].

The neurobiological mechanisms underlying the relationship between metabolic changes and mood disorders remain unclear. There are studies showing the importance of dysregulated inflammatory pathways, oxidative and nitrosative stress, disruption in the microbiota–gut–brain axis, neurotransmission imbalance and endocrine dysregulation [7,8,9,10]. In addition, it is also reported in the literature that the pathophysiology of BD is related with mitochondrial dysfunction leading to disruption of energy homeostasis [11,12].

The urge to eat is regulated by homeostatic and non-homeostatic (hedonic) processes [13]. The nucleus arcuatus in the hypothalamus plays an important role in the maintenance of energy homeostasis. There are two different neurone systems in the nucleus arcuatus that work in opposition to each other. Neuropeptide Y and agouti-related peptide (AgRP) activation increases appetite. Pro-opiomelanocortin (POMC), cocaine and amphetamine-regulated transcript peptide decrease appetite [14,15]. Neurons in this region are involved in the regulation of energy homeostasis in response to stimuli from both adipose tissue and the gastrointestinal system. Some of these hormones and neuropeptides are appetite stimulants (adiponectin, ghrelin, asprosin) and some are appetite inhibitors (leptin, nesfatin, peptide tyrosine tyrosine (PYY)) [16]. In addition to affecting the homeostatic system, nutrients also affect brain regions associated with pleasure and reward. Homeostatic and non-homeostatic (hedonic) feeding is governed by concerted action in hypothalamic and mesocorticolimbic circuits [13]. Hedonic tone is hypothesised to be closely related to mood, reward and motivation and regulated by limbic–cortical–striatal–pallidal–thalamic circuits [17]. Low hedonic tone has been associated with various psychopathologies, including depressive disorder, attention deficit hyperactivity disorder, substance use disorder and bipolar disorder [14,17].

In recent years, the role of appetite-regulating hormones in the pathogenesis and clinical progression of BD has been widely investigated [18,19]. Abnormal levels of circulating appetite hormones have been reported in patients with BD. Increased levels of adiponectin and leptin have been reported in euthymic patients with bipolar disorder-I (BD-I) compared with healthy controls [20]. In a study including a total of 57 patients with BD-I and BD-II, lower levels of glucagon-like peptide-1 (GLP-1) and ghrelin and higher levels of glucose-dependent insulinotropic polypeptide were found compared with controls and it was reported that these changes may play a role in the pathogenesis of BD [4].

Appetite hormones have been shown to play a role in various metabolic processes including insulin secretion, insulin sensitivity, energy homeostasis and inflammation as well as regulation of appetite [18,19]. Asprosin is mainly produced and released by adipocytes in white adipose tissue during fasting. Asprosin crosses the blood–brain barrier and then increases the activity of AgRP neurons in the nucleus arcuate. This signal inhibits the activity of POMC neurons in a γ-aminobutyric acid (GABA)-dependent manner, thus stimulating food intake and regulating energy homeostasis [21]. PYY is released by endocrine L cells of the colon. In humans, it causes a negative energy balance by strongly reducing food intake. The mechanism of action of PYY is based on specific activation of hypothalamic neuropeptide Y receptors leading to activation of POMC neurons. PYY is also recognised as the most potent satiety factor of the gastrointestinal system [22]. To our knowledge, no study investigating the relationship between serum asprosin and PYY levels, metabolic parameters and psychopathology in patients with BD-I has yet been conducted.

The pathophysiological basis of BD is not fully understood and there is a need to deepen the biological mechanisms underlying this complex disorder. Evaluation of the relationship between symptom severity, serum fasting asprosin and PYY levels and metabolic parameters in bipolar disorder manic (BD-M), euthymic and control subjects with BD-I may contribute to our knowledge about energy homeostasis in BD and help us understand the neurobiology of BD. We hypothesised that there would be impairments in metabolic parameters and regulation of asprosin and PYY between patients with BD-I and controls and between manic and euthymic periods of the disease. In our study, we aimed to investigate whether the clinical features, serum fasting asprosin and PYY levels and metabolic parameters of the BD-M group, euthymic group and control group differed, and to evaluate the relationship of asprosin and PYY levels with clinical features, metabolic parameters, severity of symptomatology and psychopathology in BD-I.

## 2. Participants and Methods

### 2.1. Research Sample

The patient group, consisting of 94 adults with a diagnosis of BD, included patients who applied to the psychiatry outpatient clinic of Erzurum City Hospital between November 2023 and May 2024 and/or who were treated as inpatients in our clinic. The control group consisted of 39 participants without psychiatric disorders who applied to our outpatient clinic for consultation or status report between November 2023 and May 2024. Written informed consent was obtained from all participants and/or their guardians.

The research protocol of the study was approved by the Health Sciences University Erzurum Medical Faculty Scientific Research Ethics Committee (Erzurum, Turkey) with the decision number BAEK 2023/07-89 and was conducted in accordance with the Declaration of Helsinki.

### 2.2. Procedure

Our study focused on cross-sectional clinical characteristics, routine biochemical parameters, serum asprosin and PYY analyses of 133 participants. Three distinct cohorts were incorporated into the research: the BD-M cohort (*n* = 54), the euthymic cohort (*n* = 40), and the healthy control cohort (*n* = 39). All patients were diagnosed with BD-I according to the Diagnostic and Statistical Manual of Mental Disorders-5 (DSM-5) [2]. In order to ascertain a diagnosis of BD-I, it is imperative that the individual has experienced a minimum of one manic episode during their lifetime. Episodes characterized by hypomania or major depressive states may manifest either preceding or succeeding the manic episodes [2]. The BD-M group was composed of patients who met the criteria for manic episode according to DSM-5. The euthymic cohort comprised individuals who had received a diagnosis of BD-I and who had not experienced a manic, depressive, or hypomanic episode within the preceding six-month period. The control group consisted of people without psychiatric disorders who applied to our outpatient clinic for consultation or status report.

The diagnosis of BD and exclusion of additional psychopathologies were made using clinical interview and DSM-5 clinician version (SCID-5/CV) [23]. Turkish validity and reliability study of SCID-5/CV was conducted [24]. The patients completed the Young Mania Rating Scale (YMRS) and Hamilton Depression Rating Scale (HAM-D). The control group consisted of participants without psychopathology assessed by psychiatric examination and SCID-5/CV. Participants were evaluated twice by two different psychiatrists. Clinical and sociodemographic data of the participants were recorded.

Not having an additional psychopathology beyond BD-I was determined as an inclusion criterion for the patient group. Furthermore, the inclusion criteria for participants encompassed individuals aged between 18 and 65 years, the absence of obesity (defined as BMI < 30 kg/m^2^), the absence of acute or chronic physical ailments, the absence of mental disabilities, and the exclusion of those who were pregnant or in the postpartum phase. Conversely, the presence of alcohol and substance use disorders served as exclusion criteria. Individuals with alcohol use were considered social drinkers and social drinking and tobacco use were not considered as exclusion criteria Because alcohol consumption guidelines vary significantly around the world, it was quantified as one standard drink per day for women and two standard drinks per day for men (a standardized drink is defined as containing 10−14 g of ethanol, which is equivalent to a bottle of beer (350 mL), a glass of wine (150 mL), or a shot of tequila, raki, vodka, or whiskey (44 mL)) [25,26]. Participants in the BD-M group were not undergoing pharmacological treatment at the time of their presentation to our clinic. The patients had independently ceased their medication regimens prior to this. Among the cohort, nine patients experienced their first episode of mania. The current use of antidepressant or antipsychotic medications constituted an exclusion criterion for individuals in the euthymic group, primarily due to potential metabolic side effects [27,28]. Medication status was not considered an exclusion criterion for the euthymic group due to ethical concerns and the potential risk of increased mood episodes following medication discontinuation.

The criteria for inclusion within the control group were delineated as follows: individuals must be aged between 18 and 65 years, exhibit no signs of psychopathology, possess no acute or chronic medical conditions, have no mental disabilities, maintain a body mass index below 30 kg/m^2^, and not be currently pregnant or in the postpartum phase. Furthermore, the presence of alcohol and substance use disorders constituted grounds for exclusion from the control group. However, social consumption of alcohol and tobacco use did not serve as factors for exclusion.

Characteristics of all participants, such as age, gender, education, alcohol and cigarette use, body mass index, were recorded in the sociodemographic data form. YMRS and HAM-D were filled out. YMRS was developed by Young et al. [29] and is not used to make a diagnosis, but to determine the severity of a manic state. It consists of 11 items, including elevated mood (elevated, unwarranted playfulness) and thought structure disorder (environmental thought; mildly distractible; increased thought production), and each of its questions includes five severity levels. The validity and reliability of the scale for our country has been performed. Cronbach’s alpha coefficient was found to be 0.79 [30]. The HAM-D scale was filled out to evaluate the severity of depression. This is a 17-item rating scale developed by Hamilton (1960) and filled out by clinicians [31]. The items of the scale that are related with difficulty falling asleep, waking up in the middle of the night, waking up early in the morning, somatic symptoms, genital symptoms, weakening and insight were scored between 0 and 2 and the other items were scored between 0 and 4, culminating in a total score for the scale of between 0 and 53. A score of seven and below indicates the absence of depression [32]. The validity and reliability study of the Turkish version was conducted. Cronbach’s alpha coefficient was found to be 0.75 [33].

### 2.3. Biochemical Analysis

Fasting blood samples were procured from the subjects between the hours of 08:00 and 10:00 from the antecubital vein utilizing a vacutainer. The levels of Alanine Aminotransferase (ALT), Aspartate Aminotransferase (AST), Gamma Glutamyl Transferase (GGT), glucose, total cholesterol, high-density lipoprotein cholesterol (HDL-c), low-density lipoprotein cholesterol (LDL-c), triglycerides, glycated hemoglobin (HbA1c), as well as levels of Peptide YY (PYY) and Asprosin were analyzed from the collected blood specimens. For routine biochemistry parameters, ALT, AST, and GGT activities and glucose, total cholesterol, HDL-c, LDL-c, and triglyceride levels were determined by the spectrophotometric method with a Siemens Atelica (Forchheim, Germany) clinical chemistry analyser by centrifugation after clotting for 30 min at room temperature. HbA1c levels were measured by high-performance liquid chromatography in a Lifotronik H9 device (Shenzhen, China) in whole-blood samples taken in EDTA-containing hemogram tubes. For PYY and asprosin levels, blood was collected in yellow-capped biochemistry tubes and, after clotting was completed, serum was separated by centrifugation at 3000 rpm for 10 min. Serum samples were aliquoted and frozen at −80 °C until analysed. After the serum samples were thawed under appropriate conditions, all analyses were performed in a single session in Erzurum City Hospital Medical Biochemistry Laboratory. PYY and asprosin levels in serum samples were analysed on a RelAssay automatic ELISA reader (Biobase Biodusty, Co., Ltd., Jinan, China) using BT Lab (Jiaxing, China) Elisa kits according to the standard protocol recommended by the manufacturer. The reliability of the ELISA test is ensured by keeping the false positive rate low by using antibodies with high specificity. Thanks to its high sensitivity it can detect even low concentrations of antigens or antibodies, enabling early diagnosis. The measurement range of the kit was 3–900 pg/mL and 0.5–100 ng/mL of antigens and antibodies, respectively, as specified in the data sheet. To enhance the accuracy and reliability of biochemical analyses, various quality control methods have been implemented. During laboratory processes, all data and samples were meticulously tracked using sample codes. Additionally, the performance of the devices was checked using quality control samples before each analysis session. Quality assurance was ensured by regularly calibrating the devices and using standardized protocols. The accuracy and reliability of the ELISA test kits were ensured through analyses performed with high accuracy and in accordance with the protocol provided by the manufacturer. Measurements were repeated, and consistent results were obtained, demonstrating the reliability of the data. There were no instances of missing data or of low-quality data.

### 2.4. Statistical Analysis

The study analyses were performed using IBM Statistical Package for Social Sciences (SPSS) version 22 (IBM Corp., Armonk, NY, USA). The normal distribution of continuous variables was examined by Shapiro–Wilk test, Kolmogorov–Smirnov test, Q–Q plot, skewness and kurtosis. Data were presented as mean, standard deviation, percentage and number. Comparisons between categorical variables were made using the chi-square test. For comparisons between two independent groups, the independent samples *t* test was used as the normal distribution condition was met. The one-way ANOVA test was used for comparisons of continuous variables with more than two independent groups as the normal distribution condition was met. Cohen’s d statistic was used for effect sizes between two groups and eta squared statistic was used for effect sizes between three groups. Post-hoc tests after the one-way ANOVA test were performed using Bonferroni test when variances were homogeneous and Tamhane’s T2 test when variances were not homogeneous. Correlation analysis was performed to evaluate the relationship between quantitative variables. The Pearson correlation test was used for the comparison of two quantitative variables as the normal distribution condition was met. The results of the correlation analysis are presented both as table and scatter plots. Receiver operating characteristic curve (ROC) analysis was performed to determine whether continuous variables could be used in diagnosis and cut-off values. ANCOVA was used in multiple comparisons to examine the effects of co-factors on the dependent variable. Statistical significance level was taken as *p* < 0.05.

## 3. Results

The study included 94 patients with BD-I and 39 healthy controls. Of the 94 patients included in the study, 54 met the criteria for manic episodes and 40 were in the euthymic period. Comparison of sociodemographic and clinical characteristics of the patient and control groups is shown in Table 1.

There were significant differences in BMI among the three groups. The BMI of the patients was significantly higher compared with the control group (*p*_manic-control_ = 0.012, *p* _euthymic control_ < 0.001). Triglyceride levels in the euthymic group were found to be significantly higher than those in the BD-M group (*p*_manic-euthymic_ = 0.034) and the control group (*p*_euthymic-control_ = 0.002). Glucose levels were found to be significantly higher in the BD-M group compared with the euthymic (*p*_manic-euthymic_ = 0.008) and control groups (*p*_manic-control_ < 0.001). Asprosin and PYY levels showed significant differences among the three groups (*p* < 0.001). The levels of asprosin and PYY were lowest in the BD-M group and highest in the control group. The effect size for asprosin was 0.18 (eta squared = 0.18), and for PYY it was 0.21 (eta squared = 0.21). Disease-related data between the BD-M group and the euthymic group are shown in Table 2. The relationship between clinical and blood parameters of the three groups is presented in Table 2, and the post hoc analysis results are presented in Table 3.

**Figure 1:**
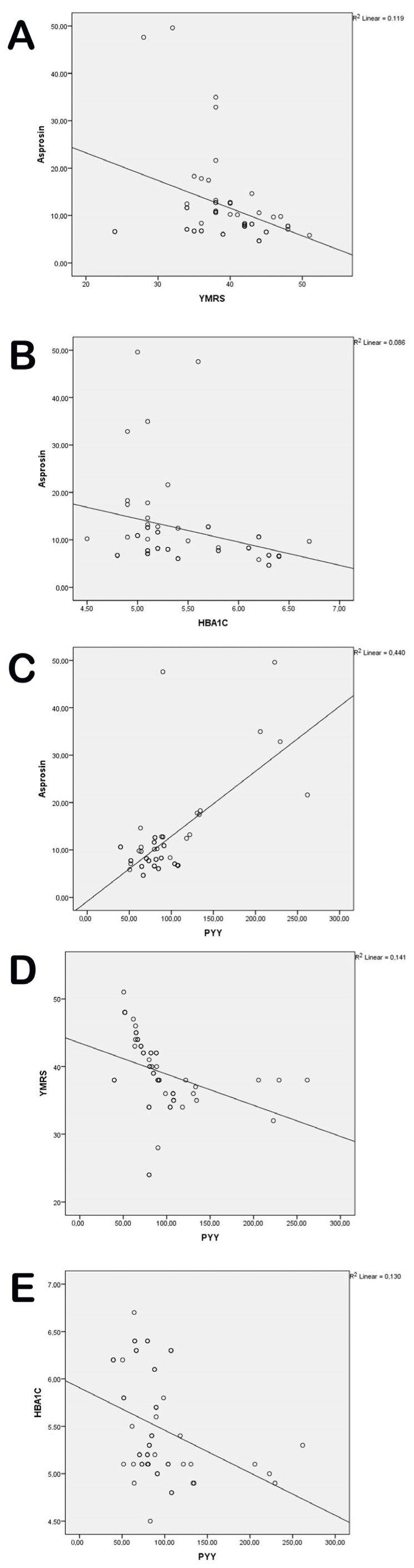
(**A**) Relationship between asprosin and YMRS in the BD-M group. (**B**) Relationship between asprosin and HbA1c in the BD-M group. (**C**) Relationship between asprosin and PYY in the BD-M group. (**D**) Relationship between PYY and YMRS in the BD-M group. (**E**) Relationship between PYY and HbA1c in the BD-M group. Abbreviations: YMRS, Young mania rating scale; HBA1C, haemoglobin A1c; PYY, peptide tyrosine tyrosine.

**Figure 2:**
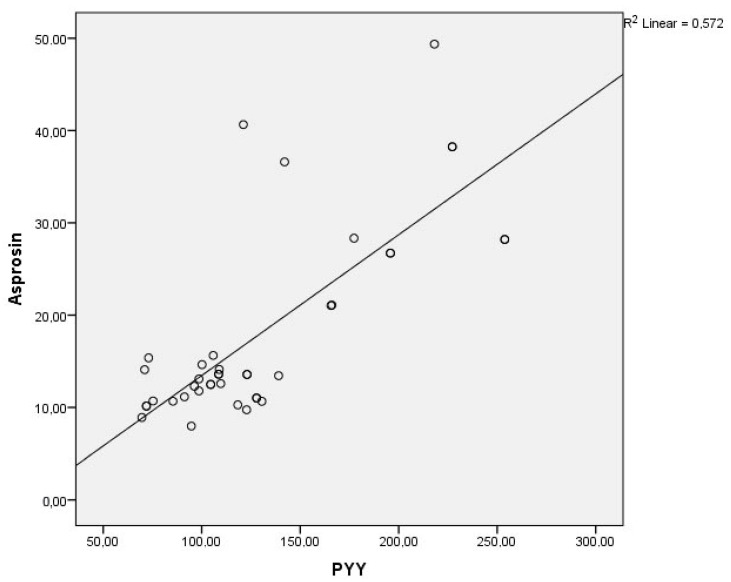
Relationship between asprosin and PYY in euthymic group. Abbreviation: PYY, peptide tyrosine tyrosine.

In the BD-M group, a moderate negative correlation was found between YMRS and asprosin levels (*r* = −0.345, *p* = 0.011) and PYY levels (*r* = −0.376, *p* = 0.005). Similarly, in the BD-M group, HbA1c levels were moderately negatively correlated with asprosin (*r* = −0.294, *p* = 0.031) and PYY levels (*r* = −0.361, *p* = 0.007) (Table 4). A strong positive correlation was found between asprosin and PYY in both the BD-M group (*r* = 0.663, *p* < 0.001) and the euthymic group (*r* = 0.756, *p* < 0.001) (Table 4, Figure 1 and Figure 2). No significant relationship was found between BMI and asprosin or PYY in any of the three groups.

**Figure 3:**
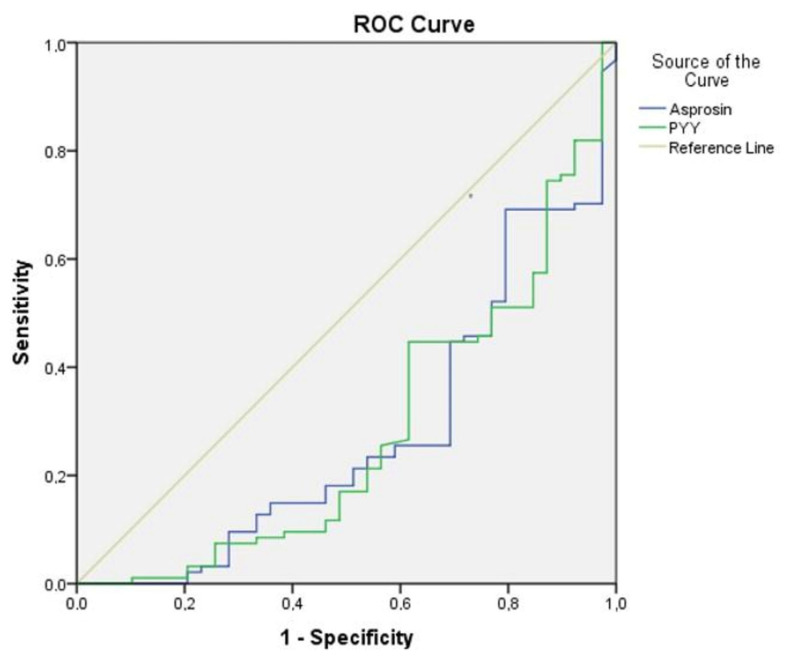
ROC curve analysis for asprosin and peptide tyrosine tyrosine (patient control group). Note: asprosin (cut-off value 14.2, sensitivity 69.2%, specificity 74.5%); PYY, peptide tyrosine tyrosine (cut-off value 122.3, sensitivity61.5%, specificity73.4%).

**Figure 4:**
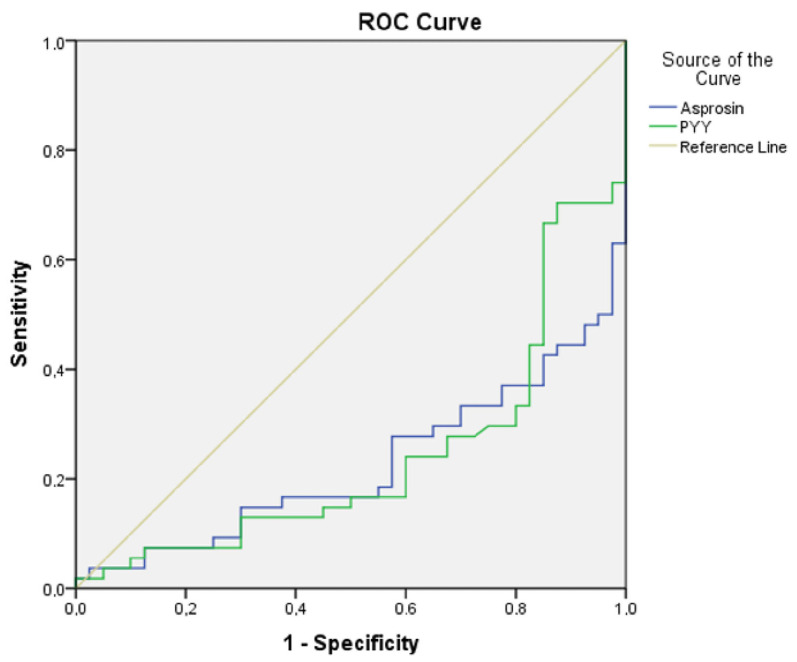
ROC curve analysis for asprosin and peptide tyrosine tyrosine (BD-M euthymic group). Note: asprosin (cut-off value 11.7, sensitivity 70%, specificity 70.4%); PYY, peptide tyrosine tyrosine (cut-off value 98.6, sensitivity 72.5%, specificity 72.2%).

ROC analysis was conducted to elucidate the relationship between asprosin and PYY concerning both the disease manifestation and manic episodes in individuals diagnosed with the condition. The findings from the analysis indicated that asprosin (AUC ± SE; 95% CI; 0.724 ± 0.049; 0.627–0.821) and PYY (AUC ± SE; 95% CI; 0.720 ± 0.051; 0.621–0.819) exhibited a significant association with BD-I. Within this context, the identified cut-off value for asprosin was determined to be 14.2 (sensitivity 69.2%, specificity 74.5%), while the cut-off value for PYY was established at 122.3 (sensitivity 61.5%, specificity 73.6%). Furthermore, the analysis results substantiated that asprosin (AUC ± SE; 95% CI; 0.775 ± 0.048; 0.682–0.869) and PYY (AUC ± SE; 95% CI; 0.760 ± 0.050; 0.662–0.865) were significantly correlated with manic episodes in BD-I. In this regard, the cut-off value for asprosin was identified as 11.7 (sensitivity 70%, specificity 70.4%), and the cut-off value for PYY was noted as 98.6 (sensitivity 72.5%, specificity 72.2%) (Table 5, Figure 3 and Figure 4).

To evaluate the differences between groups (BD-M euthymic group, BD-M control group, euthymic control group), ANCOVA was performed with asprosin and PYY separately adjusted as covariates. As there was no correlation between BMI and the dependent variable (both asprosin and PYY), BMI was not taken as a covariate. In comparing the BD-M group and the euthymic group, the difference in PYY remained significant after adjusting asprosin as a covariate (*p* = 0.040 for matched BD-M euthymic group). When asprosin was the dependent variable and PYY was adjusted as a covariate, no significant difference was found between the groups. In comparing the BD-M group and the controls, the difference in PYY remained significant after adjusting asprosin as a covariate (*p* = 0.013 for matched BD-M control group). When asprosin was the dependent variable and PYY was adjusted as a covariate, no significant difference was found between the groups. In comparing the euthymic group and the controls, the difference was not significant after adjusting both asprosin and PYY as covariates (*p* = 0.324 for matched euthymic control group). The ANCOVA analysis for asprosin and PYY (BD-M euthymic group and BD-M control group) is shown in Table 6.

## 4. Discussion

Disturbances in energy homeostasis and metabolic imbalances are commonly observed in BD patients [4,18]. We hypothesized that there would be disruptions in metabolic parameters and the regulation of asprosin and PYY between both BD-I patients and controls, and between the manic and euthymic periods of the disease. In this study, we evaluated the differences in serum asprosin and PYY levels among the BD-M group, euthymic group, and healthy controls, and assessed the relationship between asprosin and PYY levels with clinical characteristics, metabolic parameters, symptom severity, and psychopathology in BD-I. Our results show that BMI, fasting blood glucose, and triglycerides, which are precursors of metabolic disorders, had higher values in patients. The serum asprosin and PYY levels of the patients were found to be significantly lower compared with healthy controls. The lowest serum asprosin and PYY levels were observed in the BD-M group. It was shown that asprosin and PYY could be used to predict both the disease and the manic period. The difference in PYY remained significant between matched groups (BD-M euthymic, BD-M control) when the effect of asprosin was controlled.

In our study, asprosin levels showed significant differences among the three groups. The minimal concentrations of asprosin were observed in individuals experiencing the manic phase, whereas the maximal concentrations of asprosin were recorded in the healthy control group. It has been reported that asprosin increases the activity of AgRP neurons in the arcuate nucleus, regulates energy homeostasis by stimulating food intake, and promotes an anabolic state by encouraging positive energy balance of AgRP [14,21]. Decreased serum asprosin levels may lead to reduced AgRP levels, indicating a negative energy balance and catabolism [14,34]. It can be suggested that, in patients during the manic phase, impaired energy metabolism and low serum asprosin levels improve over time as patients become euthymic, approaching blood levels seen in the control group; however, the significant difference with controls persists. Therefore, low asprosin levels may be specific to both BD-I and the manic phase. Additionally, a significant negative relationship was found between disease severity according to YMRS and asprosin levels in patients during the manic phase. These results support the idea that low asprosin levels may be a characteristic specific to both the disease and the manic phase. To our knowledge, our study is the first to evaluate asprosin levels in BD-I patients and compare them with healthy controls. Parlak et al. (2016) compared the serum AgRP levels of BD-I patients with healthy controls and found that serum AgRP levels in patients during the manic phase were significantly lower compared with euthymic patients and controls [18]. The study has suggested that low serum AgRP levels could be an indicator of impaired energy homeostasis during manic episodes and that AgRP might be considered a state marker for manic episodes [18].

In our study, participants in the euthymic group were using at least one of the classic mood stabilizers such as lithium and valproic acid. The use of mood stabilizers by patients in the euthymic phase could be considered a confounding factor. However, patients in the BD-M group and the controls were not using any medication. The administration of antipsychotic and antidepressant medications constituted an exclusion criterion in the present investigation owing to their associated metabolic adverse effects [27,28]. It has been reported that antipsychotics can affect energy metabolism in patients with euthymic bipolar disorder by altering the activity of mitochondrial functions [35]. Additionally, the pathophysiology of BD has been suggested to be associated with mitochondrial dysfunction leading to impaired energy homeostasis, and lithium and valproic acid have been reported to treat mitochondrial dysfunction in BD [9,11,12,36]. In our study, the use of mood stabilizers such as lithium and valproic acid by patients in the euthymic phase could explain why the energy metabolism in this group was more stable compared with the BD-M group.

Asprosin, which plays an important role in energy homeostasis, stimulates food intake [21], while PYY is a satiety hormone and strongly reduces food intake in humans, causing a negative energy balance [22]. An interesting finding of the study is that both manic and euthymic patients had lower levels of asprosin and PYY compared with controls, and a strong positive correlation was found between the two molecules. Furthermore, dyslipidemic conditions and metabolic dysregulations were identified among the subjects in our investigation; however, no substantial correlation was established between asprosin, PYY, and the metabolic variables assessed. A significant negative relationship was found between disease severity according to YMRS and PYY levels in patients during the manic phase. These results suggest that low PYY levels may be a characteristic specific to the disease and phase. In recent years, the role of appetite-regulating hormones in the onset and clinical progression of psychiatric disorders has been widely investigated. Postprandial PYY levels have been found to be positively associated with grey matter volume and negatively associated with postprandial activity in the caudate nucleus, as well as negatively correlated with cerebral blood flow in the prefrontal and paralimbic regions involved in reward behaviour. Thus, PYY has been suggested to act centrally to modulate eating behaviour via striatal networks [37]. Gastrointestinal hormones like GLP-1 and PYY have been reported to possess receptors expressed in brain regions that regulate not only hunger and energy metabolism but also stress, behaviour, and cognitive function [38]. Lower PYY levels have been measured in the cerebrospinal fluid of patients with schizophrenia compared with controls, and it has been suggested that treatment with neuroleptics does not affect PYY levels, and low PYY levels could be used as a trait marker of the disease [39].

In the present investigation, we ascertained that serum concentrations of asprosin and PYY were markedly diminished in the BD-M cohort in comparison to both the euthymic group and the control subjects and that upon controlling for the influence of asprosin, the distinction in PYY between the corresponding groups (BD-M-euthymic, BD-M-control) persisted as statistically significant. Furthermore, we observed that diminished PYY levels exhibited a stronger correlation with BD-I and manic episodes. In contrast to GLP-1, we could not identify sufficient empirical evidence within the existing literature that directly correlates PYY with BD-I. To our knowledge, our study is the first to evaluate PYY levels in BD-I patients and compare them with healthy controls. However, considering that both neuropeptide Y and GABA are secreted by neurons in the arcuate nucleus and that GABA is inhibited by the neuropeptide Y family, the lack of GABA inhibition associated with impaired cognitive function in BD may be due to decreased peripheral PYY concentrations [40,41]. In addition to playing a significant role in energy homeostasis, appetite-regulating hormones have been reported to affect immune-inflammatory responses known to be dysregulated in BD [6,18]. The correlation between elevated glucose and lipid concentrations and a compromised immune response is a conclusion substantiated by empirical evidence in individuals experiencing their first episode of psychosis [42]. The diminished levels of asprosin and PYY documented in our investigation may signify an underlying immune-metabolic dysregulation, and the observed variability in biomarker levels during episodes of mania and periods of euthymia may be interpreted as integral components of the etiopathological framework of this disorder. In a mouse model, it was shown that butyrate and propionate stimulate the secretion of GLP-1 from enteroendocrine L cells through the free fatty acid receptor 2 [43]. In the literature, it has been reported that reduced concentrations of butyrate-producing bacteria in patients with BD may reduce GLP-1 secretion, and GLP-1 receptors are expressed in brain regions responsible for the regulation of mood and cognition, such as the cerebral cortex, hypothalamus (arcuate nucleus and ventromedial nucleus), and limbic system (amygdala and hippocampus) [38,44]. Liraglutide, a GLP-1 agonist, has been reported to improve manic-like symptoms by enhancing hippocampal oxidation and brain-derived neurotrophic factor levels in a BD model induced by D-amphetamine [45]. It was found that serum GLP-1 levels in patients with BD were significantly lower compared with healthy controls and negatively correlated with previous mood episodes [5].

This investigation encompasses both strengths and constraints. The primary constraint of the investigation resides within its cross-sectional design. Such a design restricts the capacity to deduce causal relationships between asprosin and PYY levels in relation to disease states. Additionally, the cross-sectional design fails to furnish insights regarding the temporal fluctuations of these biomarkers. Another potential constraint within our investigation pertains to the absence of concurrent radiological assessments for the participants. Other limitations include a deficiency in a more extensive examination of appetite stimulants and inhibitors. Furthermore, the dietary patterns and circadian rhythms of the participants were not extensively analysed. It warrants attention that sleep disturbances and eating disorders may correlate with metabolic irregularities in individuals with bipolar disorder, particularly during manic episodes. Another limitation arises from the fact that patients in the euthymic phase were undergoing treatment with mood stabilizers. Conversely, patients categorized within the BD-M group were not administered any pharmacological treatments. Nevertheless, we lack sufficient information regarding the medications previously utilized by these patients, including duration of use, which represents an additional limitation of the study. Moreover, although the anorectic properties of PYY are predominantly ascribed to PYY3-36, our study exclusively quantified total PYY concentrations. The distinct receptor selectivity profiles of the two subtypes, PYY1-36 and PYY3-36, may have influenced the outcomes of the research [37]. Although our sample size was deemed adequate, it is crucial to acknowledge that the study did not encompass a more extensive sample population, thus rendering the findings derived from a singular centre non-generalizable. Conversely, our investigation unveiled significant findings, such as diminished serum asprosin and PYY levels in BD-I patients relative to control subjects, with the most pronounced reductions observed in the BD-M group, suggesting that PYY may serve as a status marker for manic episodes, alongside elevated levels of BMI, fasting blood sugar, and triglycerides—precursors to metabolic disorders—in BD-I patients. Additionally, the incorporation of diagnostic assessments grounded in structured clinical interviews, the validated reliability of the scales employed in the Turkish context, the dual evaluation of participants by independent psychiatrists, the comprehensive consideration of multiple variables alongside asprosin and PYY, and the selection of an appropriately matched control group constitute further strengths of the study.

Although our findings indicate substantial disparities, it is imperative to validate our conclusions through longitudinal, comparative investigations that assess various phases of the disorder and its correlation with other psychiatric conditions. Subsequent research that investigates the energy and reward pathways in conjunction with larger sample sizes and multicentre collaborations may enhance our comprehension of the neurobiological mechanisms underlying BD-I, facilitate early identification of manic episodes, and aid in the monitoring of mood fluctuations. Furthermore, it is recommended that these biomarkers be appraised as potential therapeutic targets for the regulation of circadian rhythms and the management of metabolic disorders.

## 5. Conclusions

Our results show that serum asprosin and PYY levels were significantly lower in BD-I patients compared with healthy controls. The lowest serum asprosin and PYY levels were found in the BD-M group. Despite patients showing higher BMI, dyslipidemia, and metabolic imbalances compared with controls, there was no significant correlation between asprosin and PYY levels and metabolic parameters. Additionally, a negative relationship was found between disease severity according to YMRS and asprosin and PYY levels in patients during the manic phase. A positive correlation was observed between asprosin and PYY in both manic and euthymic patients. These findings suggest that low asprosin and PYY levels may be indicators of impaired energy homeostasis in BD-I. Moreover, PYY may serve as a state marker for manic episodes.

## Figures and Tables

**Table 1:** Comparison of demographic variables between the three groups.

		BD-M Group *n*%	Euthymic Group *n*%	Control Group *n*%	Chi-Square/F	*p*
Age (year)	Mean ± SD	37.56 ± 13.22	39.88 ± 9.87	38.69 ± 11.53	0.446	0.641
Gender	Female	31 (57.4)	21 (52.5)	15 (38.5)	3.355	0.187
	Male	23 (42.6)	19 (47.5)	24 (61.5)		
Marital status	Married	27 (50)	19 (47.5)	25 (64.1)	5.127	0.266
	Single	23 (42.6)	15 (37.5)	13 (33.3)		
	Widowed, divorced, living apart	4 (7.4)	6 (15)	1 (2.6)		
Occupation	Unemployed	39 (72.2)	21 (52.5)	1(2.6)	68.523	<0.001
	Private sector employee	7 (13)	10 (25)	2(5.1)		
	Public employee	8 (14.8)	9 (22.5)	36 (92.3)		
Educational status	Primary school	22 (40.7)	16 (40)	2 (5.1)	53.864	<0.001
	High school	28 (51.9)	17 (42.5)	8 (20.5)		
	University	4 (7.4)	7 (17.5)	29 (74.4)		
Smoking	Yes	26 (48.1)	5 (12.5)	17 (43.6)	14.006	0.001
	No	28 (51.9)	35 (87.5)	22 (56.4)		
Alcohol use	Yes	2 (3.7)	0 (0)	5 (12.8)	5.905	0.028
	No	52 (96.3)	40 (100)	34 (87.2)		
Presence of mental illness in the family	Yes	4 (7.4)	9 (22.5)	4 (10.3)	5.011	0.082
	No	50 (92.6)	31 (77.5)	35 (89.7)		

*p* < 0.05: statistical significance level; BD-M group: bipolar disorder manic group; F: one-way ANOVA test value; Mean ± SD: mean ± standard deviation; *n*: number of participants.

**Table 2:** Comparison of clinical characteristics and blood parameters of groups.

	BD-M Group *n* = 54 Mean ± SD	Euthymic Group *n* = 40 Mean ± SD	Control Group *n* = 39 Mean ± SD	t/F	Effect Size/Eta Squared	*p*
Duration of illness (year)	11.33 ± 9.34	14.33 ± 9.21	-	1.545	0.32	0.126
Total number of episodes	6.00 ± 4.76	4.25 ± 2.75	-	−243	0.45	0.027
Number of manic episodes	3.70 ± 2.45	2.53 ± 1.43	-	−2.921	0.58	0.004
Number of depressive episodes	2.30 ± 2.78	1.73 ± 1.71	-	−1.229	0.24	0.222
YMRS	39.15 ± 5.49	3.90 ± 2.54	-	−41.543	8.24	<0.001
HAM-D	1.06 ± 1.22	2.63 ± 1.90	-	4.566	0.98	<0.001
BMI (kg/m^2^)	27.73 ± 2.04	29.12 ± 0.83	26.26 ± 2.57	21.001	0.24	<0.001
Triglyceride (mg/dL)	123.76 ± 64.78	156.63 ± 58.19	113.13 ± 49.85	6.012	0.08	0.003
HDL-c (mg/dL)	43.48 ± 13.89	38.18 ± 12.41	40.89 ± 9.74	1.444	0.02	0.240
LDL-c (mg/dL)	112.66 ± 43.25	121.99 ± 28.09	121.36 ± 36.55	0.942	0.01	0.393
Total Cholesterol (mg/dL)	160.09 ± 37.37	175.03 ± 37.69	170.82 ± 36.80	2.030	0.03	0.135
ALT (U/L)	24.22 ± 12.31	22.28 ± 13.81	24.95 ± 10.78	0.503	0.01	0.606
AST (U/L)	24.04 ± 12.76	15.85 ± 7.24	13.87 ± 4.44	15.754	0.19	<0.001
GGT (U/L)	22.61 ± 13.51	29.50 ± 16.96	22.72 ± 14.95	2.898	0.04	0.059
Glucose (mg/dL)	102.13 ± 22.16	88.03 ± 21.56	82.77 ± 11.09	12.628	0.16	<0.001
HbA1c (%)	5.49 ± 0.56	5.29 ± 0.51	5.33 ± 0.38	1.997	0.03	0.140
Asprosin (ng/mL)	12.05 ± 9.28	17.93 ± 10.16	26.29 ± 18.40	13.918	0.18	<0.001
PYY (pg/mL)	94.01 ± 44.85	130.12 ± 51.60	172.88 ± 90.43	17.597	0.21	<0.001

*p* < 0.05: statistical significance level; ALT: alanine aminotransferase; AST: aspartate aminotransferase; BD-M group: bipolar disorder manic group; BMI: body mass index; effect size = Cohen’s d (0.2—small effect size, 0.5—medium effect size, and 0.8—large effect size); eta squared: 0.01—small effect size, 0.06—medium effect size, and 0.14—large effect size; F: one-way ANOVA test value; GGT: gamma glutamyl transferase; HAM-D: Hamilton depression rating scale; HbA1c: glycosylated haemoglobin; HDL-c: high-density lipoprotein-cholesterol; LDL: low-density lipoprotein-cholesterol; Mean ± SD: mean ± standard deviation; *n*: number of participants; PYY: peptide tyrosine tyrosine; t: independent samples *t* test, YMRS: Young mania rating scale.

**Table 3:** Posthoc analyses results.

	*p* Value		
	BD-M Group Euthymic Group	BD-M Group Control Group	Euthymic Group Control Group
Age (year)	1	1	1
BMI (kg/m^2^)	<0.001	0.012	<0.001
Triglyceride (mg/dL)	0.034	0.754	0.002
HDL-c (mg/dL)	0.293	0.966	1
LDL-c (mg/dL)	0.505	0.653	1
Total Cholesterol (mg/dL)	0.172	0.521	1
ALT (U/L)	1	1	1
AST (U/L)	0.001	<0.001	0.380
GGT (U/L)	0.090	1	0.141
Glucose (mg/dL)	0.008	<0.001	0.442
HbA1c (%)	0.248	0.295	0.983
Asprosin (ng/mL)	0.015	<0.001	0.046
PYY (pg/mL)	0.002	<0.001	0.037

*p* < 0.05: statistical significance level; ALT: alanine aminotransferase; AST: aspartate aminotransferase; BD-M group: bipolar disorder manic group; BMI: body mass index; GGT: gamma glutamyl transferase; HbA1c: glycosylated haemoglobin; HDL-c: high-density lipoprotein-cholesterol; LDL-c: low-density lipoprotein-cholesterol; PYY: peptide tyrosine tyrosine.

**Table 4:** Relationship of asprosin and peptide tyrosine tyrosine with clinical characteristics and blood parameters of patients in the BD-M and euthymic groups.

		BD-M Group		Euthymic Group	
		Asprosin	PYY	Asprosin	PYY
Age	*r*	−0.173	−0.096	−0.301	−0.248
	*p*	0.211	0.489	0.059	0.122
BMI	*r*	−0.059	−0.237	−0.045	−0.165
	*p*	0.670	0.084	0.782	0.310
Duration of illness	*r*	−0.157	−0.067	−0.165	−0.309
	*p*	0.257	0.632	0.309	0.052
Total number of episodes	*r*	−0.181	−0.090	0.082	−0.137
	*p*	0.190	0.516	0.615	0.398
Number of manic episodes	*r*	−0.148	−0.037	0.068	−0.147
	*p*	0.286	0.788	0.676	0.364
Number of depressive episodes	*r*	−0.179	−0.122	0.075	−0.098
	*p*	0.195	0.381	0.646	0.548
YMRS	*r*	−0.345	−0.376	−0.053	0.010
	*p*	0.011	0.005	0.745	0.949
HAM-D	*r*	0.275	−0.017	−0.118	−0.016
	*p*	0.044	0.904	0.469	0.923
Triglyceride	*r*	−0.042	0.219	0.132	0.031
	*p*	0.766	0.112	0.415	0.850
HDL-c	*r*	0.097	0.088	−0.203	−0.109
	*p*	0.484	0.527	0.208	0.504
LDL-c	*r*	−0.186	−0.192	0.062	0.211
	*p*	0.178	0.165	0.705	0.192
Total Cholesterol	*r*	−0.204	−0.086	−0.001	0.085
	*p*	0.139	0.536	0.994	0.601
ALT	*r*	−0.195	−0.153	0.056	−0.132
	*p*	0.157	0.270	0.730	0.416
AST	*r*	−0.014	0.076	0.167	0.052
	*p*	0.922	0.583	0.304	0.749
GGT	*r*	0.132	0.136	0.054	−0.036
	*p*	0.341	0.327	0.742	0.825
Glucose	*r*	0.159	0.180	−0.075	−0.308
	*p*	0.249	0.192	0.647	0.053
HbA1c	*r*	−0.294	−0.361	−0.009	−0.118
	*p*	0.031	0.007	0.955	0.469
Asprosin	*r*	1	0.663	1	0.756
	*p*		0.000		0.000
PYY	*r*	0.663	1	0.756	1
	*p*	0.000		0.000	

*p* < 0.05: statistical significance level; ALT: alanine aminotransferase; AST: aspartate aminotransferase; BD-M group: bipolar disorder manic group; BMI: body mass index; GGT: gamma glutamyl transferase; HAM-D: Hamilton depression rating scale; HbA1c: glycosylated haemoglobin; HDL-c: high-density lipoprotein-cholesterol; LDL-c: low-density lipoprotein-cholesterol; PYY: peptide tyrosine tyrosine; YMRS: Young mania rating scale.

**Table 5:** Area under the curve.

Test Result Variable(s)		Area	Std. Error ^a^	Asymptotic Sig. ^b^	Asymptotic 95% Confidence Interval	
					Lower Bound	Upper Bound
Patient group (total) Control group	Asprosin	0.724	0.049	0.000	0.627	0.821
	Peptide tyrosine tyrosine	0.720	0.051	0.000	0.621	0.819
Test Result Variable(s)		Area	Std. Error ^a^	Asymptotic Sig. ^b^	Asymptotic 95% Confidence Interval	
					Lower Bound	Upper Bound
BD-M group Euthymic group	Asprosin	0.775	0.048	0.000	0.682	0.869
	Peptide tyrosine tyrosine	0.760	0.050	0.000	0.662	0.859

*p* < 0.05; Statistical significance level; BD-M group: bipolar disorder manic group; ^a^ Under the nonparametric assumption; ^b^ Null hypothesis: true area = 0.5.

**Table 6:** ANCOVA for asprosin and peptide tyrosine tyrosine.

BD-M Group Compare with Euthymic Group					
Dependent variable: asprosin					
Source	df	SS	MS	F	Sig.
Covariate (PYY)	1	4427.155	4427.155	96.70	0.000
Group	1	8.422	8.422	0.18	0.669
Error	91	4166.17	45.782		
Total	94	29,287.874			
Dependent variable: PYY					
Source	df	SS	MS	F	Sig.
Covariate (asprosin)	1	108,430.276	108,430.276	96.70	0.000
Group	1	4864.763	4864.763	4.34	0.040
Error	91	102,038.203	1121.299		
Total	94	1,365,019.696			
BD-M group compare with Control group					
Dependent variable: asprosin					
Source	df	SS	MS	F	Sig.
Covariate (PYY)	1	9459.339	9459.339	106.74	0.000
Group	1	95.225	95.225	1.07	0.303
Error	90	7976.085	88.623		
Total	93	52,225.977			
Dependent variable: PYY					
Source	df	SS	MS	F	Sig.
Covariate (asprosin)	1	226,450.505	226,450.505	106.74	0.000
Group	1	13,585.579	13,585.579	6.40	0.013
Error	90	190,942.359	2121.582		
Total	93	2,060,267.896			

*p* < 0.05: statistical significance level; BD-M group: bipolar disorder manic group; PYY: peptide tyrosine tyrosine; SS: Type III sum of squares; MS: mean square.

## Data Availability

All data generated or analysed during this study are included in this article. The data will be available upon reasonable request (contact persons: filiz.mercantepe@saglik.gov.tr).
